# A Six Week Therapeutic Ballet Intervention Improved Gait and Inhibitory Control in Children With Cerebral Palsy—A Pilot Study

**DOI:** 10.3389/fpubh.2019.00137

**Published:** 2019-06-25

**Authors:** Kimberley D. Lakes, Kelli Sharp, Marybeth Grant-Beuttler, Ross Neville, Fadia Haddad, Rachel Sunico, Daniel Ho, Melinda Schneider, Sofia Sawitz, Janine Paulsen, Kim Caputo, Kim D. Lu, Afshin Aminian, Citlali López-Ortiz, Shlomit Radom-Aizik

**Affiliations:** ^1^Pediatric Exercise and Genomics Research Center, School of Medicine, University of California, Irvine, Irvine, CA, United States; ^2^Department of Psychiatry and Neuroscience, School of Medicine, University of California, Riverside, Riverside, CA, United States; ^3^Department of Dance, Claire Trevor School of the Arts, University of California, Irvine, Irvine, CA, United States; ^4^Department of Physical Medicine, School of Medicine, University of California, Irvine, Irvine, CA, United States; ^5^Crean School of Health and Behavioral Science, Chapman University, Orange, CA, United States; ^6^Center for Sports Studies, School of Public Health, Physiotherapy, and Sports Science, University College Dublin, Dublin, Ireland; ^7^School of Medicine, University of California, Irvine, Irvine, CA, United States; ^8^Pacific Coast Center for the Arts, Mission Viejo, CA, United States; ^9^Children's Hospital of Orange County, Orange, CA, United States; ^10^Neuroscience Program, Departments of Kinesiology and Community Health and Dance, University of Illinois at Urbana-Champaign, Champaign, IL, United States

**Keywords:** pediatric, rehabilitation, dance, exercise-medicine, executive functions, arts, physical activity, cerebral palsy

## Abstract

Children with cerebral palsy (CP) have motor impairments that make it challenging for them to participate in standard physical activity (PA) interventions. There is a need to evaluate adapted PA interventions for this population. Dance can promote coordination, posture, muscle strength, motor learning, and executive functioning. This pilot study evaluated the feasibility and the effects of a new therapeutic ballet intervention specifically designed for children with CP.

**Methods:** Eight children with CP (9–14 y/o; 75% female) participated in a 6-week therapeutic ballet intervention. Outcomes were measured in multiple domains, including body composition (DXA), muscle strength (hand-grip dynamometer), habitual physical activity, gait and selective motor control functions, and executive functioning. Follow-up assessments of habitual physical activity, gait, and executive functioning were completed 4 to 5 weeks post-intervention.

**Results:** Five of the eight participants were overfat or obese based on DXA percentage of body fat. All participants were below the 50th percentile for their age and gender for bone density. Four participants showed a trend to improve hand-grip strength in one hand only, while one improved in both hands. There were significant improvements in gait across time points (pre, post, and follow-up), specifically in time of ambulation (*X*_pre_ = 4.36, *X*_post_ = 4.22, *X*_follow−up_ = 3.72, *d* = 0.056, *p* = 0.02), and in step length (cm) on the right: *X*_pre_ = 48.29, *X*_post_ = 50.77, *X*_follow−up_ = 52.11, *d* = 0.22, *p* = 0.027, and left stride: *X*_pre_ = 96.29, *X*_post_ = 102.20, *X*_follow−up_ = 104.20, *d* = 0.30, *p* = 0.027, indicating gait changes in bilateral lower extremities. There was improvement in inhibitory control (*d* = 0.78; 95% Confidence Limit = ±0.71, *p* < 0.05) with large individual responses primarily among those above the mean at baseline.

**Conclusions:** Therapeutic ballet may prove to be a useful intervention to promote physiological and cognitive functions in children with CP. Results demonstrated feasibility of the physical, physiological, and cognitive assessments and suggested improvements in participants' gait and inhibitory control with large individual responses. Modifications to personalize the intervention may be needed to optimize positive outcomes.

**Clinical Trial Registration:** www.ClinicalTrials.gov, identifier: NCT03681171

## Introduction

Cerebral palsy (CP) is a neurobiological disorder that is induced by injury to the brain during prenatal and early postnatal development. CP is characterized by disturbance in movement that is usually accompanied by impairments in cognitive and behavioral functions. There is evidence suggesting that early intervention with children with CP may promote functional connectivity in the brain, which may in turn improve prognosis over time (1, 2); thus, interventions during childhood should be a high priority for CP research.

It is well established that physical activity (PA) interventions can improve both motor and cognitive functions in healthy children (3) and children with special needs (4). Among the various physical activity interventions that have been previously studied in children with CP, therapeutic ballet, which combines body movements and artistic expression appears to be a promising practice (5). In a pilot study that assessed the perceived benefits of a ballet intervention for children with CP, children reported enjoyment and interest in continuing ballet classes, and their parents perceived therapeutic benefit (5). Therapists involved in the study perceived benefits beyond traditional physical therapy in self-confidence, in the use of the body as instrument for creative expression, in the exposure to music and rhythm to guide movement, and in providing an alternative structure for physical rehabilitation interventions. The balletic foundation of this therapeutic intervention was theoretically supported, in part, by the modular and hierarchical organization of the conservatory style instruction of ballet technique, which has been in development during the last five centuries. This type of instructional organization allows for rationally organized motor learning, promoting a larger and more complete repertoire of available motor actions (5). The effectiveness of this modular approach to improve balance was tested in a randomized controlled clinical trial in children with CP (ages 7–15 y/o) consisting of a 12 h of targeted ballet in a studio over a 4 week period. This study reported improvements post-intervention in the Pediatric Balance Scale for the ballet group that were retained at 1 month follow-up (6).

Although disruptions in motor functioning are often the most recognizable feature of CP, executive function impairment is also a central feature of CP (7). Recent studies have indicated that dance may be an example of a cognitively complex and social physical activity that could be expected to improve executive functions (8, 9). Thus, in the current pilot study, we aimed to study the impact of a therapeutic ballet intervention on motor and executive functioning. We characterized anthropometric, body composition, bone health, muscle strength, and habitual physical activity in a group of children with CP and evaluated for the first time the effects of a 6-week therapeutic ballet intervention on gait, motor, and executive functioning.

## Methods

### Participants

Eight children with CP (9–14 y/o; 75% female) completed a 6-week (three times per week) therapeutic ballet intervention that included live piano music. The inclusion criteria were: (1) cerebral palsy spastic diplegia and/or hemiplegia; (2) absence of health problems that would preclude participation in exercise; (3) male or female age 9–14 years; (4) ability to read and complete study measures; (5) ability to participate in dance classes conducted in English; (6) interest in learning ballet; (7) intact vision; (8) ability to ambulate independently in the community with or without a device; (9) intact proprioception in lower extremities; and (10) ability to complete assessment measures and consents in English. Five of the participants had a history of prematurity (26–34 weeks gestation), one participant had a history of congenital heart defect (double outlet right ventricle), and one participant was born full term with microcephaly of unclear etiology. The Institutional Review Board at the University of California Irvine approved the study, and written informed consent and assent were obtained from all participants and their guardians upon enrollment.

### Assessment Procedures

Physical, physiological and cognitive assessments were conducted in two sessions before and after the ballet intervention. Session one included clinical, anthropometric, body composition, muscle strength and cognitive (executive functioning) assessments and was completed in the PERC Human Performance Laboratory. Session two included an evaluation of gait and physical functioning in a movement laboratory. In addition, habitual physical activity was assessed over 7 days during the first and last weeks of the intervention. A follow-up assessment with a subset of measurements was conducted 4 to 5 weeks after the post-intervention assessment and included measures of motor function, habitual physical activity, and executive functioning.

### Measures

#### Anthropometric Measurements

Standard calibrated scales and stadiometers were used to measure height and body mass. Body mass index [BMI = wt/ht^2^ (kg/m^2^)] percentile and stature percentile were calculated using the online calculators from the Centers for Disease Control (CDC) (10–12).

#### Body Composition and Bone Health

Body composition and bone density were determined by a Dual X-ray Absorptiometry (DXA), using Hologic QDR 4500 densitometer. Participants were scanned in light clothing while lying supine. The DXA instrument was calibrated using the procedures provided by the manufacturer, and DXA scans were performed and analyzed using a pediatric software. Percent fat categories were calculated based on body fat reference curves for children (13). The 2nd, 85th, and 95th percentiles define the cut off points for underfat, overfat, and obese.

#### Hand Grip Strength

Grip strength was measured using a Jamar handgrip dynamometer, which evaluated maximum isometric strength (Kg) of the hand and forearm muscles. The participants sat comfortably in a chair with a back support, with elbow at 90°, and squeezed the dynamometer with maximum isometric effort that was maintained for 3 to 5 s. No other body movements were allowed. Three trials, with brief pauses, were done for each hand alternately. The best result at each assessment was chosen for analyses. Participants were strongly encouraged to exert maximum effort. Results are presented as absolute values in kg and relative to norms of healthy boys and girls of the same age (14).

#### Selective Control Assessment of the Lower Extremity (SCALE)

To assess the selective voluntary motor control [SVMC; (15)] participants were asked to perform specific isolated movements at each joint to bilaterally assess the movements of the hip, knee, ankle, subtalar, and toe joints. Evaluation was performed in the sitting position, except for assessing hip flexion, which is tested in side-lying for proper joint excursion. For each joint, the participants were asked to perform a task by moving their limbs through the desired movement sequence using a 3-s verbal count. The SCALE score for each limb is the sum of all the points given to each joint, up to a maximum of ten points per limb.

#### Gait Function

The GAITRite© system (16) was used to collect temporal and spatial gait parameters. Each participant ambulated for a total of four trials at each testing time point. The GAITRite is an 8.3 m long and 0.89 m wide carpet that forms an electronic walkway with pressure sensors embedded into the carpet in a horizontal grid. As the participant walks over the carpet mat, the sensors are triggered when mechanical pressure is applied (17). The active area of the mat is ~7.32 m long and 0.61 m wide. A distance of 12.7 mm separates the sensors between one another. The data was sampled at a frequency of 80 Hz, allowing a temporal resolution of 11 ms from the carpet mat runway (16). Each participant ambulated barefoot for a total of four trials at each testing time point. As defined by the GAITRite system (16), stride length is measured on the line of progression between the heel points of two consecutive footprints of the same foot. Step length is determined by the distance from the heel center of the current footprint to the heel center of the previous footprint on the opposite foot.

#### Executive Functions: Hearts and Flowers Tasks

The Hearts & Flowers EF tasks (18, 19) are tasks that measure executive functions, including attention, inhibitory control, working memory, and shifting/cognitive flexibility. These tasks were administered to participants individually by a researcher using a touch-screen laptop computer. All participants completed the task on the same laptop computer and placed their hands in the same position on a resting bar in front of the screen in order to maintain equal distances from the screen across all participants. Completion of the task (consisting of three blocks of trials) required about 7 min per child, including set-up, instructions, and practice. In the first block (Congruent), children were asked to press a button on the screen on the same side as the stimulus (a heart). Following practice, there are 12 trials in which the heart appears on either the left or right side of the screen. For the second block (Incongruent), children were asked to press the button on the opposite side of the stimulus (a flower). After practice, there were 12 trials. The final block (Mixed) consisted of 33 trials in which the child is presented with either a heart or flower; children are required to inhibit automatic responses, retain rules in working memory, and quickly shift between rules to press the correct button. Performance was evaluated using accuracy scores and median response time (in milliseconds) for each of the three blocks. The first trial of each block was omitted from analysis for both accuracy and response time scores. Accuracy scores were based on the percentage of correct responses in a given block (number of correct responses divided by the number of trials). To reduce the effects of outliers, a median response time was calculated for each block (18); analysis is based on response times for correct responses.

#### Habitual Physical Activity

Activity monitors (Actigraph GT3X) were worn on the waist during awake time during the first and last weeks of the ballet intervention and at the time of the follow up visit 4 to 5 weeks after the completion of the intervention. Twelve hour daytime activity data was analyzed for 3–4 weekdays using Actilife software. Physical activity was classified into sedentary, light, moderate, and vigorous levels according to the cut-points set by Mattocks Children 2007 (20). Cut points based on vector magnitude <100 counts per minute (CPM) were scored as sedentary, 3581–6129 CPM were scored as moderate, and >6130CPM were scored as vigorous physical activity. Results are presented as moderate, vigorous and moderate to-vigorous physical activity (MVPA).

### Intervention

Following the baseline assessments, participants attended 1-h ballet sessions three times per week for 6 weeks. Sessions were held in a university dance studio. Make up sessions were held to ensure all participants attended a minimum of 16/18 h of the intervention. Licensed physical therapists and experienced dance teachers delivered the ballet intervention. Physical therapy and dance students also were present to assist the children, resulting in a minimum of a 1:1 child to assistant ratio. The assistants provided physical support as needed, to help the child initiate and perform the dance steps. Each participant wore a gait belt during all weight bearing activities to enhance overall safety.

The adapted ballet classes mirrored components of typical ballet classes, including warm-up, stretching, barre exercises, and center floor exercises, with reverence to finish each class. Specific exercises addressed CP-related gait issues, such as out-of-pattern movement, postural alignment, endurance in sitting and standing, weight shifting, and out-of-phase motion in lower extremities. Engaging exercises promoted selective motor control, balance, and coordination. Each class was augmented with live piano music and prop targets (such as wands, bean bags, hula hoops, chalk, and stickers) to improve motor approximation and maintain participants' attention and motivation. For novelty and challenge, as participants repeated and learned steps, the next session's exercises increased in difficulty, adding more complex steps and combinations. The intervention concluded with a class demonstration for parents and members of the community where the children's accomplishments were celebrated.

#### Exercise Intensity During Ballet Sessions

Intensity of the activities during two of the ballet sessions was assessed using Polar E600 heart rate monitors. Minimum, maximum, and average HR (bpm) during the ballet sessions are reported as means ± standard deviations.

### Analyses

To analyse data collected to characterize participants, scores were generated according to measurement protocols and were evaluated for change over the three time points. Paired *t*-tests were used to evaluate changes in BMI percentile, percent body fat, bone density, and grip strength (using the significance level of *p* < 0.05). Repeated measures ANOVA was used to evaluate moderate-to-vigorous physical activity (MVPA) over the three time points.

Primary outcomes were analyzed using several methods selected based on their appropriateness for the data, study design, and sample size. Data from the SCALE was analyzed using a Fisher's Exact Test in R (21). Gait function data was analyzed with GraphPad Prism (La Jolla, CA) and Practical Meta-Analysis Effect Size Calculator (22) using Friedman's test (significant *p* < 0.05) to determine effect size for specific gait parameters (using means and Cohen's *d*).

Data from the executive functions computer tasks were analyzed in SAS Studio using a mixed linear model (Proc Mixed). A multi-level growth model was used, where repeated measurements were nested within individual subjects and where change over time was specified to vary across participants (23). Effects (Cohen's *d*) were derived via standardization: i.e., mean change over time divided by the standard deviation at baseline. A process outlined in Hopkins (24) and Joseph et al. (25) was implemented to remove the small sample-size bias in these estimates of standardized effects. Inferences were derived using a magnitude-based inference (MBI) approach to likelihood estimation. This approach [outlined in Hopkins et al. (26)] is a supplement to null-hypothesis testing and a refinement of Cohen's *d*. It ensures that the outcomes were interpreted according to both statistical significance and the likelihood that they were a substantial-sized effect. The size of standardized effects were evaluated according to the following scale: <0.2, trivial; 0.2–0.6, small; 0.6–1.2, moderate; >1.2, large (26, 27). The accompanying magnitudes express the likelihood of these effects and are presented as qualitative probabilistic inferences according to the following scale: 25–75%, possibly (^*^); 75–95%, likely (^**^); 95–99%, very likely (^***^); >99.5%, most likely (^****^). Uncertainty in the estimate is expressed using 95% confidence limits.

Due to the lack of available control group data in this study, further precision of the estimate was achieved by estimating the presence and extent of (i) regression toward the mean and (ii) individual responses and responders in significant outcomes. The process for calculating (i) and (ii) followed the steps outlined in Hopkins (28–30), respectively. Reliability data from the control group in Schonert-Reichl et al. (31) were obtained and used to facilitate these estimations.

## Results

### Participant Characteristics

#### Anthropometric, Percent Body Fat, and Bone Health

Table 1 presents anthropometric characteristics, percent body fat, and bone health of the eight participants. Based on BMI percentile, five participants had normal weight, one was overweight (90th percentile), and two were underweight (1st percentile). Based on percent body fat measured by DXA, three participants were obese, two overfat, and three normal fat. All participants were below the 50th percentile for their age and gender for bone density. No significant changes were observed in BMI percentile, percent body fat, or bone density from before to after the intervention.

**Table 1:** Baseline anthropometric, percent body fat, and bone density data.

**Age range (years)**	**Height (cm)**	**Weight (Kg)**	**BMI %ile**	**Stature for age %ile**	**% Fat (DXA)**	Fat category^*^ (based on % fat)	Total body BMD^**^z score	**Total hip BMD z score**	**Total spine BMD *z* score**
12–14	160.7	59	90	76.9	29.9	Obese	−1.1	−0.8	0.4
9–11	139.4	34	47	17.8	35.7	Obese	−0.7	−1.7	−2.2
9–11	132.7	26	14	24.9	34.3	Obese	−2.4	−2.9	−2.1
12–14	156.7	45	33	13.5	18.9	Normal	−0.1	−1.3	−0.7
9–11	137.1	28	16	50.2	30.7	Overfat	−1	−0.2	−1.5
9–11	134.7	25	1	9.9	24.4	Normal	−2.3	−1.4	−1.8
9–11	123.1	20	1	0.8	29.1	Overfat	−3	−4.6	−1.1
9–11	142.53	34	38	75.7	27.1	Normal	−0.7	−1.4	−0.7

* *The 2nd, 85th, and 95th percentiles define the cut offs for underfat, overfat, and obese (13)*.

** *BMD, Bone Mineral Density*.

#### Habitual Physical Activity

On average, in the first week of the intervention, participants spent 44 ± 22 min/day on moderate physical activity and 21 ± 10 min on vigorous physical activity (65 ± 28 min/day MVPA), with no significant change at the end of the intervention (60 ± 22 min/day MVPA) and 4–5 weeks following the intervention (58 ± 24 MVPA).

#### Muscle Strength

At baseline, six participants had asymmetric right/left hand grip strength (Table 2). On average the group performed 61% ± 33 of predicted values on the right hand and 53% ± 25 of predicted values on the left hand. Four participants showed a trend to improve handgrip strength in one hand only, while one improved in both hands.

**Table 2:** Hand grip strength at baseline and post intervention.

**Age range (years)**	**Dominant hand**	**Baseline right hand (Kg)/(% predicted)**	**Post right hand (Kg)/(% predicted)**	**Baseline left hand (Kg)/(% predicted)**	**Post left hand (Kg)/(% predicted)**
12–14	Right	32/(120)	30/(112)	14/(56)	13/(52)
9–11	Right	12/(53)	14/(68)	12/(53)	10/(44)
9–11	Right	9/(56)	9/(56)	6/(40)	5/(33)
12–14	Left	34/(97)	31/(58)	28/(96)	30/(102)
9–11	Right	12/(75)	11/(69)	4/(27)	5/(33)
9–11	Left	7.5/(33)	9/(44)	15/(73)	16/(78)
9–11	Right	10/(44)	8/(35)	2/(10)	2/(10)
9–11	Left	2/(9)	1/(4)	14/(68)	16/(78)

#### Intervention Adherence

All 8 participants adhered to the intervention and participated in at least 16 out of the 18 intervention sessions.

#### Heart Rate (HR) Evaluation During the Ballet Sessions

The average HR recording time was 54 ± 6 min per session. Average HR during the session was 97 ± 10 bpm ranging from 74 ± 10 to 133 ± 15 bpm and reflects light aerobic activity.

### Primary Outcomes

#### Selective Control Assessment of the Lower Extremity (SCALE)

**Figure 1:**
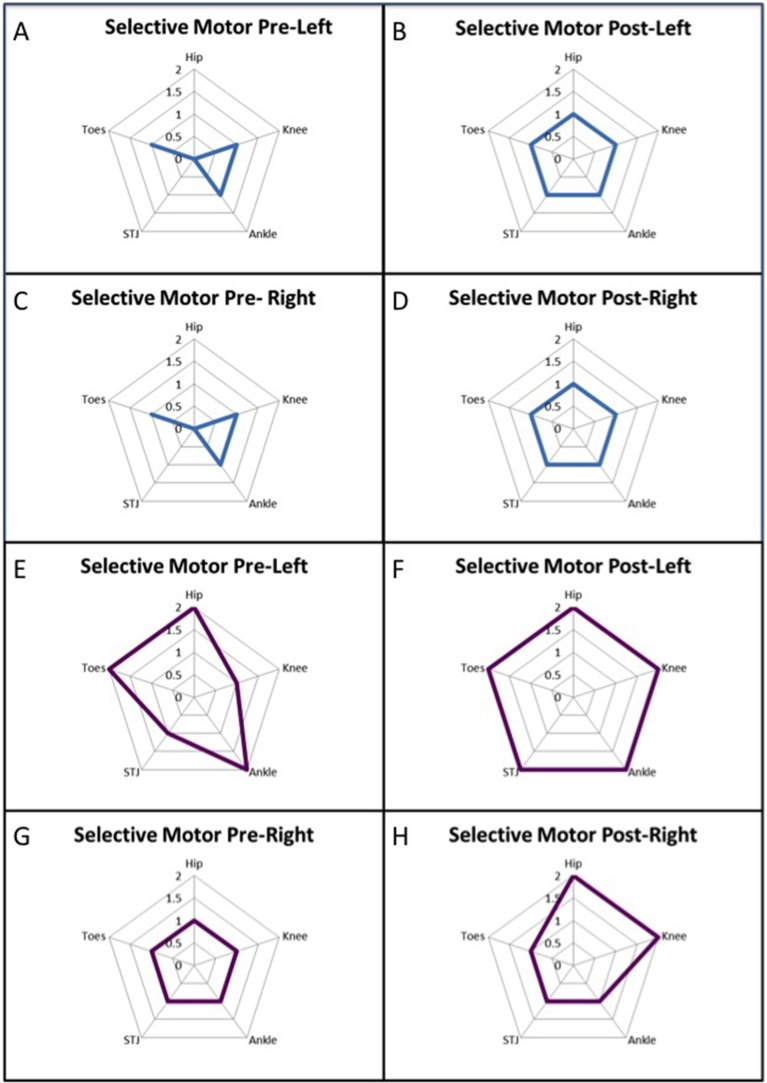
Data from two participants who demonstrated increased total limb score for the Selective Control Assessment of the Lower Extremity (SCALE). **(A–D)** illustrate the participant improved in bilateral hip and subtalar joint (STJ), where as in panels **(E–H)** a different pattern of improvement was observed in the left knee and subtalar joint and right hip and knee.

There were no significant group changes in the selective control at any joint. Changes were noticed in individual participants (see Figure 1). It is noteworthy to mention, that 62.5% of the participants improved on their total limb score unilaterally or bilaterally with majority of the improvements observed in the hip and ankle.

#### Gait

There were significant differences between pre, post, and follow-up assessments of time of ambulation (*p* = 0.02). The participants were able to ambulate with a decreased time after the intervention and follow-up testing sessions (*X*_pre_ = 4.36, *X*_post_ = 4.22, *X*_follow−up_ = 3.72, *d* = 0.056). There was a trend across all time points that the participants exhibited increased velocity. There were significant differences in the percentage of gait cycle (*p* = 0.008, *X*_pre_ = 36.50, *X*_post_ = 38.23, *X*_follow−up_ = 38.76, *d* = 0.41) spent in left single support, indicating more time was spent executing gait function on the left after participating in the intervention. There were significant differences in step length (cm) on right (*p* = 0.027, *X*_pre_ = 48.29, *X*_post_ = 50.77, *X*_follow−up_ = 52.11, *d* = 0.22) and stride length (cm) on left (*p* = 0.027, *X*_pre_ = 96.29, *X*_post_ = 102.20, *X*_follow−up_ = 104.20, *d* = 0.30), indicating gait changes in bilateral lower extremities. It is noteworthy to mention that there also was a positive trend in increased stride length on the right (*p* = 0.052, *X*_pre_ = 95.74, *X*_post_ = 102.50, *X*_follow−up_ = 103.20, *d* = −0.34).

#### Executive Functioning

The Hearts and Flowers test has three tasks (congruent, incongruent, and mixed) that yield six scores (a response time and an accuracy score for each of the three tasks). The results of these three tasks and six scores are summarized in Table 3.

**Table 3:** Pre- and Post-intervention executive function scores with Magnitude-based Inferences (MBI) for the standardized difference (Cohen's *d*) in means.

		**Baseline mean ± SD *n* = 9**	**Follow-up mean ± SD *n* = 9**	**Cohen's *d* [95% CI]**	***p*-value**	**Inference**
**Inhibitory control, working memory, and cognitive flexibility (hearts and flowers computer task)**						
Congruent trial (hearts)	Accuracy	87.96 ± 21.29	87.50 ± 11.79	0.02 [−0.22, 0.26]	0.86	Trivial^***^
	Response time	797.56 ± 142.89	798.25 ± 148.00	0.02 [−0.63, 0.65]	0.95	Unclear
Incongruent trial (flowers)	Accuracy	52.77 ± 23.20	71.86; ± 24.37	0.78 [0.07, 1.49]	0.03	Moderate^***^
	Response time	903.11 ± 183.79	745.86 ± 111.87	0.89 [0.44, 1.33]	<0.001	Moderate^****^
Mixed trial (hearts and flowers)	Accuracy	51.52 ± 19.64	58.44 ± 21.12	0.38 [0.01, 0.75]	0.05	Small^**^
	Response time	886.67 ± 144.83	916.29 ± 109.39	0.12 [−0.43, 0.67]	0.64	Unclear

Inferences were derived by means of standardization (mean difference divided by the between-groups SD at baseline). Magnitude-based inferences were evaluated according to a revision of Cohen's d: <0.2, trivial; 0.2–0.6, small; 0.6–1.2, moderate; >1.2, large (26, 27). CI, Confidence interval. Asterisks indicate effects clear at the 95% level and likelihood that the true effect is substantial, as follows:

** *likely*,

*** *very likely*,

**** *most likely (26)*.

Results for the incongruent task indicate that participants were more accurate (Cohen's *d* = 0.78; 95% Confidence Limits = ±0.71) and had faster response times (0.89; ±0.45) at post-test. These results were both statistically significant (*p* < 0.05) and substantial, with outcomes for accuracy and response time very likely and most likely moderate in terms of their effect size, respectively. Results for the mixed task indicate that the group were also more accurate upon retest. This change in mixed accuracy, while statistically significant (*p* = 0.047), was likely small (0.38 ± 0.37) in terms of its effect size. All other outcomes were either trivial or trivial-sized and unclear and not statistically significant and are, therefore, not presented here.

*Post hoc* analyses were conducted to (i) assess the presence and extent of regression toward the mean in substantial effects, (ii) estimate the presence and extent of individual responses to the intervention and (ii) identify individual responders. A lack of available reliability data for response time trials meant that the *post-hoc* analyses are only conducted on accuracy outcomes.

**Figure 2:**
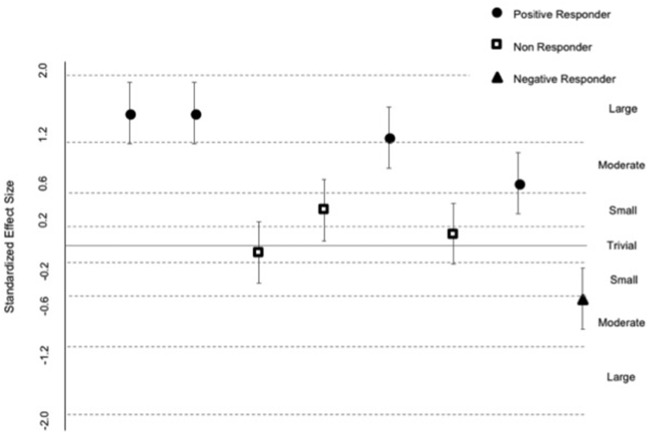
Standardized effects for individual responders. Data are individual participant change scores. Uncertainty in each change score (95% Confidence Limits) was derived by multiplying the standard error of measurement from a reliability study (31) by the t-statistic produced in the mixed model (Proc Mixed) [see (30)]. Standardized effects were derived by dividing each change score and its CL by the baseline standard deviation for incongruent trial accuracy. The size of the effects are evaluated against the following scale: <0.1, trivial; 0.1–0.2, small; 0.2–0.6, moderate; 0.6–1.2, large; >1.2, very large (26).

(i) There was evidence of substantial regression toward the mean in both the incongruent and mixed accuracy trial. That is, once the change scores were corrected for the presence of statistical artifact, the substantial large moderating effect of baseline scores was removed. Follow-up estimates of effect were calculated for participants at −1 standard deviation (SD) below and +1 SD above the mean at baseline to further assess the effects of the intervention. These outcomes indicate that it was most likely only participants at +1 SD above the mean on incongruent trials at baseline whose accuracy improved. Whilst these outcomes were not statistically significant (*p* = 0.06), they were likely moderate (1.01 ± 1.06) when assessed via MBI estimation. Outcomes for participants at −1 SD below the mean at baseline on incongruent accuracy, whilst small-sized (0.34 ± 1.09), were neither statistically significant (*p* > 0.05) nor clear when assessed via MBI. Outcomes for participants at −1 SD below (0.22 ± 1.52) and +1 SD above (0.12 ± 1.42) the mean at baseline on mixed accuracy trials were neither statistically significant nor clear.

(ii) The standard deviation representing individual responses in the incongruent accuracy trial was very large (*d* = 0.93; 95% Confidence Interval = 0.24, 1.29). This outcome is corroborated by the estimates derived for individual responders illustrated in Figure 2. Figure 2 indicates that there were four clear positive, one clear negative, and three non-responders to the intervention. Of the positive responders, there were three large responders and one moderate responder. The negative response was moderate-sized, and the estimates for the three non-responders were unclear. These estimates for individual responders with confidence limits are summarized for each participant in Table 4.

**Table 4:** Individual incongruent trial change scores with 95% confidence limits for the standardized difference (Cohen's *d*) in mean.

**ID**	**Change score [95% CI]**	**Cohen's *d* [95%CI]**	**Response**	**Size**
**Incongruent trial (flowers)—accuracy**				
1	46.72 [35.91, 57.53]	1.68 [1.29, 2.06]	Positive	Large
2	46.97 [36.16, 57.78]	1.69 [1.30, 2.07]	Positive	Large
3	−2.26 [−13.07, 8.55]	−0.08 [−0.47, 0.31]	Non	Unclear
4	12.63 [1.82, 23.44]	0.45 [0.07, 0.84]	Non	Unclear
5	38.14 [27.33, 48.95]	1.37 [0.98, 1.76]	Positive	Large
6	4.30 [−6.51, 15.11]	0.15 [−0.23, 0.54]	Non	Unclear
7	22.22 [11.41, 33.03]	0.80 [0.41, 1.19]	Positive	Moderate
8	−18.69 [−29.50, 7.88]	−0.67 [−1.06, −0.28]	Negative	Moderate

*For inferences, see Table 3*.

## Discussion

This study is the first to evaluate the effect of a therapeutic ballet intervention on gait, motor and executive functions in a group of children with CP. It is well established that physical activity (PA), a critical concern in the care of children with CP, is important for physical health. Hillman et al. hypothesized that PA in childhood could optimize brain development, but that for children with disabilities, the greatest challenge may be how to increase their PA (32). Plowman argued that PA is critical during childhood when brains are highly plastic, and that this is especially true for children with disabilities (33). However, motor impairment in children with CP appears to make participation in PA more challenging. As scientific evidence highlights the importance of PA for both motor and cognitive development (especially the development of executive functions), there is a critical need to develop and study PA interventions designed specifically for children with CP.

Verschuren et al. (34) reviewed PA and exercise intervention research for children and adolescents with CP, focusing on cardiovascular fitness using aerobic training, anaerobic training, lower-extremity muscle strength and mixed training, and found that most of the 20 studies they identified had been observational, with only five published randomized clinical trials (RCTs) at the time. Sample sizes across studies were relatively small, ranging from 3 to 46. Overall, these exercise interventions (most lasting 6 to 8 weeks) have been shown to improve a variety of outcomes, including muscle strength and tone, gross motor function, social acceptance, academic performance, posture, body image, and aerobic capacity. A pilot study for children with CP (ages 7–15) reported improvements in the Pediatric Balance Scale following 12 ballet sessions that was retained at 1 month follow-up (6). Our study extends prior research by examining the feasibility and outcomes of a therapeutic ballet intervention designed to meet the needs of children with CP (ages 9–14) on motor functioning, gait, and executive functions.

### Physical and Physiological Characteristics of Participants (Tables 1, 2)

As previously reported in children with CP (35), most of the participants in our study presented abnormalities in growth (stature for age: 0.8–76.9%ile) and body composition (Table 1). While based on BMI percentile, five participants had normal weight, one was overweight (90th percentile) and two were underweight (1st percentile), a DXA scan revealed that most participants were overfat or obese based on percent body fat (including one of the participants with a BMI in the 1st percentile), indicating the importance of evaluating body composition in a clinical setting. All participants were below the 50th percentile for their age and gender for bone density suggesting poor bone density content. Poor bone density is common in children with moderate to severe CP and increases the risk of painful, pathological fractures. Interestingly, higher body fat has also been identified as one of the risk factors for fractures in this population (36). We found no significant changes in BMI percentile, percent body fat, or bone density from before to after the intervention. However, a longer duration of the intervention may be more likely to benefit both body composition and bone health (37, 38).

To assess habitual physical activity (HPA), defined as any bodily movement in daily life which results in energy expenditure, all participants were given accelerometers to wear on a belt around the waist during wakening time for 7 days. On average, in the first week of the intervention participants spent 65 ± 28 min per day on MVPA, which is 52 ± 25 percent of the time observed in typically developing children of the same age and sex (39). Our results match data presented in a systematic review of physical activity and sedentary behavior in CP which reported that across all ages and levels of motor function, young people with CP participated in 13–53% less HPA than their typically developing peers (40). In a review that included 10 studies with children with CP (mean age of 8.4 years), Keawutan et al. indicated that all but one study showed a direct association between motor ability and HPA (41). There was no significant change in MVPA time at the end of our ballet intervention and 4–5 weeks following the intervention, indicating that the brief ballet intervention in and of itself was not enough to promote HPA. A longer intervention and/or more specific instructions and education on HPA during leisure time might better promote HPA in this population.

Hand-grip dynamometer is a simple and easily accessible way to assess muscle weakness in children with CP, and the overall reliability is considered to be good (42). Muscle strength measured by hand-grip dynamometer was 61% ± 33 of predicted values on the right hand and 53% ± 25 of predicted values on the left hand, with great variability among participants and dominant and non-dominant hands (Table 2). Four participants showed a trend to improve hand-grip strength in one hand only, while one improved in both hands. While maximal grip strength can provide valuable insight into the maximal strength of the muscle groups involved, there is a need to perform other assessments as well to evaluate muscle coordination and endurance, which are important for the performance of skilled manual tasks in daily activities of children with CP.

### Improvements in Selective Control and Gait

It is well established that individuals with CP, a non-progressive neurological disorder, have impaired motor function and selective motor control, but it is important to note that it is not an unchangeable condition with respect to gross motor and selective motor control (43, 44). Increased ambulation speed, increased left single limb support, increased stride length on the left and step length on the right, all suggested an improved ability to coordinate the out of phase or alternating control between the limbs. In addition, these improvements may be the result of the intervention's focus on weight shifting and selective movement of the ankle and foot in single limb stance practice. Improvements observed in the SCALE data suggest improved selective voluntary motor control, which could improve gait speed and control and is consistent with previous correlations between SCALE data and movement during gait (15). Shuman et al. recently published further support for the ability for enhanced gait function, with increased gait speed and gait parameters in children with CP with standard of care (45).

### Improvements in Executive Functions (Inhibitory Control)

Although disruptions in motor functioning are often the most recognizable feature of CP, executive function impairment is also a central feature of CP. Bodimeade et al. compared executive functions (EF) in children with CP (mean age 11 y/o) to typically developing controls (7). The performance of children with CP was worse in all areas of EF measured, including attentional control, cognitive flexibility, goal setting, and information processing. Bottcher et al. evaluated EF in children with spastic CP and found impairments in sustained and divided attention, inhibitory control, shifting, and general executive functioning (46). They noted that these impairments were associated with social and learning problems. Bottcher noted that there is extensive developmental research indicating that children's activities and play are critical to social and cognitive development, and as children with CP may have difficulties (motor or other) that restrict participation, it results in limited opportunities for learning and cognitive development (47).

PA interventions, particularly cognitively engaging interventions, can improve EF in children (3, 48). Thus, PA interventions for children with CP could simultaneously target motor and executive functioning. Evidence indicates that not all forms of exercise benefit EF equally (3, 9, 49, 50), and that “*the degree to which the exercise requires complex, controlled, and adaptive cognition and movement may determine its impact on EF”* (51). Diamond argued that moving without thought produces little change in executive functions and that practices like yoga, martial arts, and dance—which require both thought and movement—are likely to have a stronger positive effect on EF (8), and recent research has suggested that dance—or music and movement—interventions can be beneficial for other clinical populations of children (52).

Thus, one of the aims of the current study was to evaluate the impact of a dance intervention on EF in children with CP. The only EF task with substantial improvement after intervention was the Incongruent Accuracy task, which measures primarily inhibitory control (i.e., the ability to focus on relevant simuli in the presence of irrelevant stimuli) and revealed large individual responses. Those who were above average at baseline improved substantially, but there was little improvement among those below the group average at baseline. This likely reflects differences in impairment in this group of children and suggests that much greater tailoring of the intervention may be required for those who start with greater impairment in EFs. Physical education research has highlighted the importance of differentiation, which refers to the process of assessing a learner's starting point and to adapting or modifying tasks in order to ensure that each individual progresses (53, 54). In future studies, different lengths and type of exercises should be evaluated to gain more information that can be translated to personalize prescriptions with a goal to improve the benefits for individuals with varying levels of impairment.

### Conclusion

Therapeutic ballet may prove to be a useful intervention to promote gait and inhibitory control in children with cerebral palsy. Overall, results indicated improvements in participants' gait and inhibitory control with large individual responses. Modifications to the intervention might be needed on individualized basis to optimize health benefits, particularly for those with a greater level of initial impairment. This study provides support for further research, including the development of a randomized intervention study to systematically evaluate the effects of ballet intervention on physical, physiological, and cognitive functions in children with CP.

## Ethics Statement

The study was approved by the Institutional Review Board (ethics committee) of the University of California, Irvine.

## Author Contributions

KS, MG-B, SS, JP, AA, KC, and CL-O: conception, design, delivery of the 6 week intervention. KLa, KS, MG-B, SS, and SR-A: conception and design of the research study. KLa, KS, FH, MG-B, KLu, and SR-A: data collection. KLa, KS, MG-B, RN, FH, KLu, and SR-A: data analysis and interpretation. KLa, KS, RN, SS, CL-O, and SR-A: drafting the manuscript. KLa, KS, MG-B, FH, CL-O, and SR-A: critical revision of the manuscript. KLa, KS, MG-B, RN, FH, SS, JP, KC, KLu, AA, CL-O, and SR-A: approval of the final version. RS, DH, and MS add to both data collection and data analysis.

### Conflict of Interest Statement

The authors declare that the research was conducted in the absence of any commercial or financial relationships that could be construed as a potential conflict of interest.
